# Pneumomediastinum in MDA5+ Dermatomyositis: A Case Series

**DOI:** 10.31138/mjr.060124.pim

**Published:** 2024-05-21

**Authors:** Ramaswamy Subramanian, Rupal Prasad, Mamadapur Mahabaleshwar, Anjum Siddiqui, Digvijay Ekbote, Urmila Dhakad

**Affiliations:** 1 Department of Clinical Immunology and Rheumatology, JSS Medical College and Hospital, JSS Academy of Higher Research, Mysore, India,; 2 Department of Clinical Immunology and Rheumatology, KGMU, Lucknow, India

**Keywords:** dermatomyositis, MDA 5+, pneumomediastinum

## Abstract

MDA5+ DM, or anti-melanoma differentiation-associated gene 5 antibody-positive dermatomyositis (DM), is a rare autoimmune illness that primarily affects women of Asian origin. The typical presentation of MDA5+ DM includes a variety of cutaneous lesions accompanied by either no muscular weakness (amyopathic) or hypomyopathic features. In patients with MDA5+ DM, rapid progression of interstitial lung disease is a frequent manifestation associated with poor prognosis. Pneumomediastinum, spontaneous intramuscular haemorrhage, and macrophage activation syndrome form a spectrum of rare manifestations associated with MDA5+ DM. Since these issues are uncommon but fatal, it’s important to explore the approaches for diagnosis, treatments, and possible mechanisms, that are useful for prompt treatments and management of the patient.

## INTRODUCTION

Pneumomediastinum (PNM), also known as Hamman’s Syndrome, is characterised by free air pathologically invading the mediastinum. The patient may experience dysphagia, neck pain, dyspnoea, and chest pain; sub-cutaneous emphysema can occur frequently as well. However, because these symptoms can occasionally be vague, patients with mild manifestations may confuse them for other illnesses and neglect to visit a doctor, or doctors may misdiagnose or fail to diagnose these patients. As a result, the true incidence can be underestimated. An association of pneumomediastinum and melanoma differentiation-associated protein 5 (MDA 5) dermatomyositis has been rarely described. Here we describe three such cases.

## CASE SERIES

### Case 1

**Figure 1.**
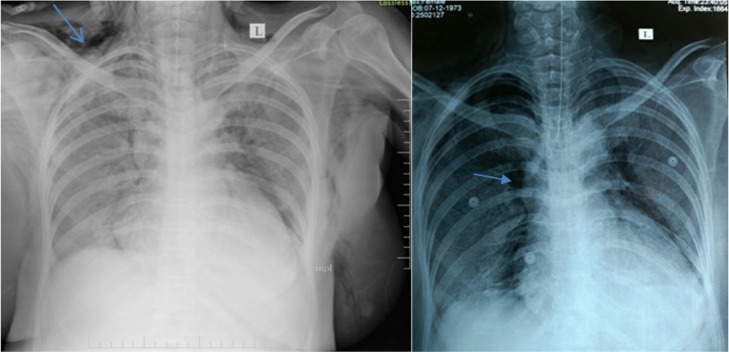
Chest X-ray showing subcutaneous emphysema and pneumomediastinum.

**Figure 2.**
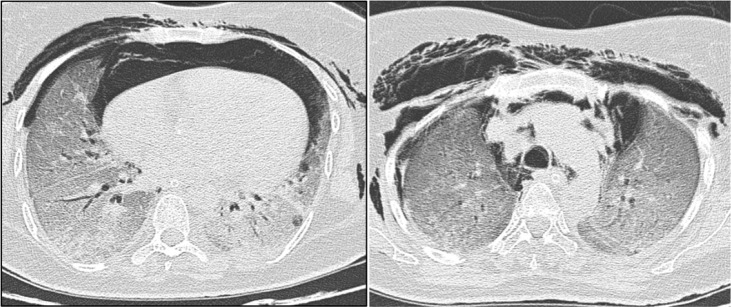
Diffuse intra-muscular, inter-muscular, and subcutaneous emphysema along with extensive GGOs in B/L lung fields.

A 52-year-old female with a background history of bronchial asthma presented with a 1-month history of polyarthralgia, easy fatiguability, and new onset dyspnoea of Modified Medical Research Council (MMRC) grade III which was rapidly progressive with no connective tissue disease (CTD)-related history. On examination, she had B/L diffuse coarse crept. She was found to be COVID-19 RTPCR + and Sputum culture showed growth of Pseudomonas and fungal hyphae. The initial CT scan had interstitial lung disease (ILD) with superimposed consolidation. The patient was started on NIV, High-Flow Nasal Oxygen (HFNO), IV Steroids, antibiotics, and antifungals. The patient continued to worsen and hence was evaluated for aetiologies of underlying ILD. She was found to have antinuclear antibodies (ANA) (by immunofluorescence [IF]) 1:160 as Nuclear Granular and extractable nuclear antigen (ENA) have positivity for SS-A (Ro) and Ku, serum ferritin was 1185ng/mL which rose to 5737ng/mL later. Incidentally, her creatinine kinase (CK) was 564IU/L. Hence, the possibility of idiopathic inflammatory myopathy (IIM) spectrum disorders was kept. Myositis-specific autoantibodies (MSA) profile was positive for MDA5. Considering her progressively worsening respiratory state she was started on intravenous immunoglobulin (IVIG), Inj Cyclophosphamide, and tab Tacrolimus. On repeat imaging her chest x-ray showed a pocket of air on the right side of the base of the neck along with translucency around cardiac borders (**Figure 1**) and her CT Chest showed extensive ILD along with pneumomediastinum and pneumothorax (**Figure 2**). However, despite therapy, she deteriorated, was shifted to mechanical ventilation, and tried extracorporeal membrane oxygenation (ECMO) but the patient died despite best efforts.

### Case 2

**Figure 3.**
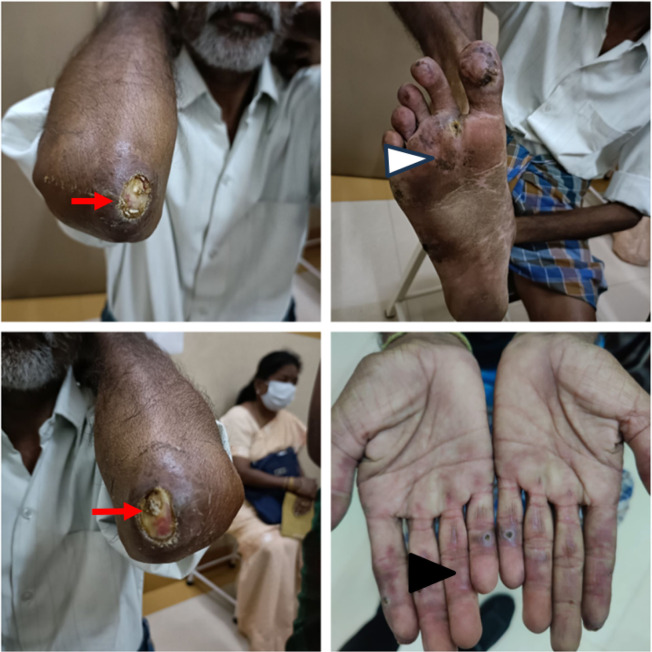
Red arrow: ulcerated lesions on bony prominences; white arrowhead: vasculitic rash; black arrowhead, inverse Gottron’s sign.

**Figure 4.**
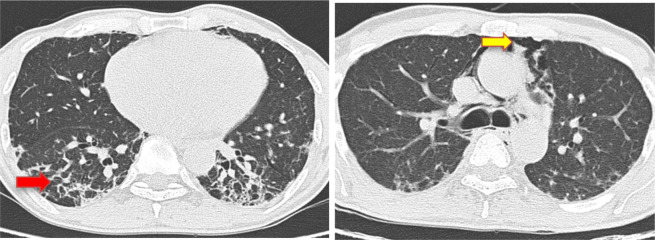
Red arrow highlighting ILD; yellow arrow showing pneumomediastinum.

A 50-year-old male presented with 2-month history of Raynaud’s phenomenon along with ulcers over his elbow, feet, and hands. He also noticed diffuse myalgia in the bilateral thigh and had significant weight loss. He also noticed shortness of breath on exertion which progressed from grade II to grade III without orthopnoea or paroxysmal nocturnal dyspnoea (PND) in a matter of 2 months. On examination he had inverse Gottron sign, palmar papules, erythematous lesions over hands, and vasculitis ulcers involving B/L elbow and feet (**Figure 3**). There was no muscular weakness. His ENA turned out to be SS-A (Ro 52) 3+, dsDNA1+, antineutrophilic cytoplasmic antibody (ANCA) profile and antiphospholipid antibody (APLA) panel was negative, normal complements, AST/ALT/LDH in normal range, normal inflammatory markers with CK NAC of 24 IU/L. Nail fold capillaroscopy showed enlarged capillaries and CECT thorax was s/o early ILD with diffuse pneumomediastinum and pneumothorax (**Figure 4**). The MSA panel is suggestive of MDA5+. The patient was managed with injectable steroids, tofacitinib, and cyclophosphamide but no significant improvement was noted. He was lost for follow-up and later expired.

### Case 3

**Figure 5.**
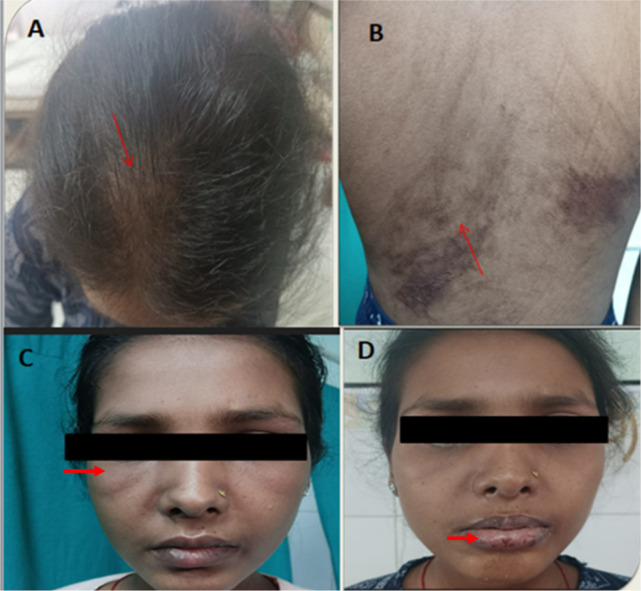
**(A)** alopecia, **(B)** flagellate erythema, **(C)** periorbital puffiness, **(D)** haemorrhagic crust over lips.

An 18-year-old female presented with complaints of fever and inflammatory polyarthritis for 9 months followed by a history of proximal muscle weakness for 2 months without bulbar or respiratory weakness. There was no history of photosensitivity, Raynaud’s phenomenon, oral ulcer, skin thickening, sicca symptoms, cough, or pedal oedema. On examination, there was periorbital oedema, erythematous crusting lesions over the lower lip, diffuse nonscarring alopecia and flagellate erythema over the trunk, Manual Muscle Test-8 (MMT 8) of 50/80, and multiple active joints (**Figure 5**). On further evaluation her ESR was 79mm/hr, serum glutamic oxaloacetic transaminase (SGOT) 429 U/L serum glutamic pyruvic transaminase (SGPT) 162 U/L, serum ferritin 400ng/ml, ANA negative, ENA RO 52 3+, and MSA panel was positive for MDA 5.

**Figure 6.**
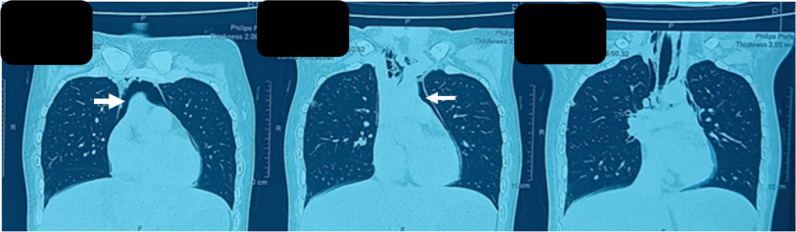
Pneumomediastinum with normal lung parenchyma (coronal section).

**Figure 7.**
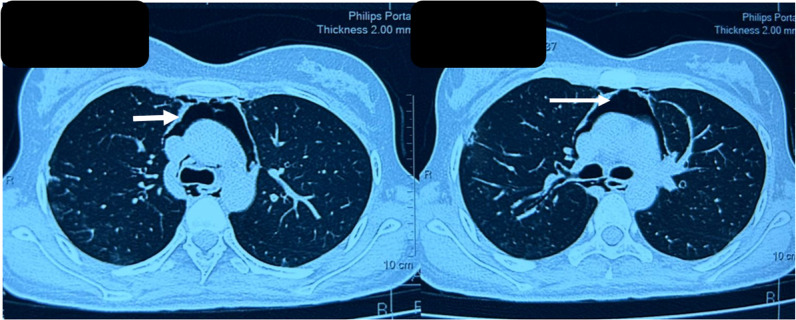
Pneumomediastinum in CECT thorax (transverse view).

The patient was started on 1 mg/kg steroid & Oral Methotrexate 15 mg/week, the patient subsequently improved in a follow-up visit with MMT8 Of 58/80. During her 3rd visit after 1-month follow-up, she developed complaints of acute onset dysphagia and substernal chest pain along with dyspnoea without any worsening in muscle weakness. Her room air saturation was 92%. Her chest X-ray, ECG, and Echo were normal while contrast enhanced computerised tomography (CECT) thorax revealed the presence of pneumomediastinum (**Figures 6–7**). She was continued on the same dose of immunosuppression and was provided with supplemental oxygen through the mask at 10L/min. She subsequently improved and was discharged with stable vitals.

## DISCUSSION AND REVIEW

Anti-melanoma differentiation-associated protein 5 (anti-MDA5) is seen in 10–35% of cases of dermatomyositis and it is an independent risk factor for interstitial lung disease-related mortality. Although clinical amyopathic dermatomyositis (CADM) is the typical presentation, 42.9–54.5% of anti-MDA5 patients present with clinically detectable myopathy. Rapidly progressive interstitial lung disease (RP-ILD) is frequently associated, and pneumothorax is a rare complication. Prevalence of ILD in dermatomyositis (DM) patients ranges from 19.9 to 78% with presentation ranging from fulminant to subclinical.^1^ Pneumomediastinum is characterised by air around the mediastinal structures with a reported prevalence of 2.2–8.3 % in all myositis cases increasing mortality rate up to 25% within the first month of diagnosis. Pneumomediastinum is a life-threatening complication reported in up to 15% of patients with anti-MDA5 DM and ILD, as compared to 2% in DM/PMN. Early diagnosis and prompt intervention are a must in MDA5-associated spontaneous pneumothorax considering the fact that mortality in cases with and without spontaneous pneumomediastinum (SPN) has been reported to be 60% and 37%, respectively.^2^ Pneumomediastinum has been reported with other CTDs also, IIM being the most frequent cause.

**Figure 8.**
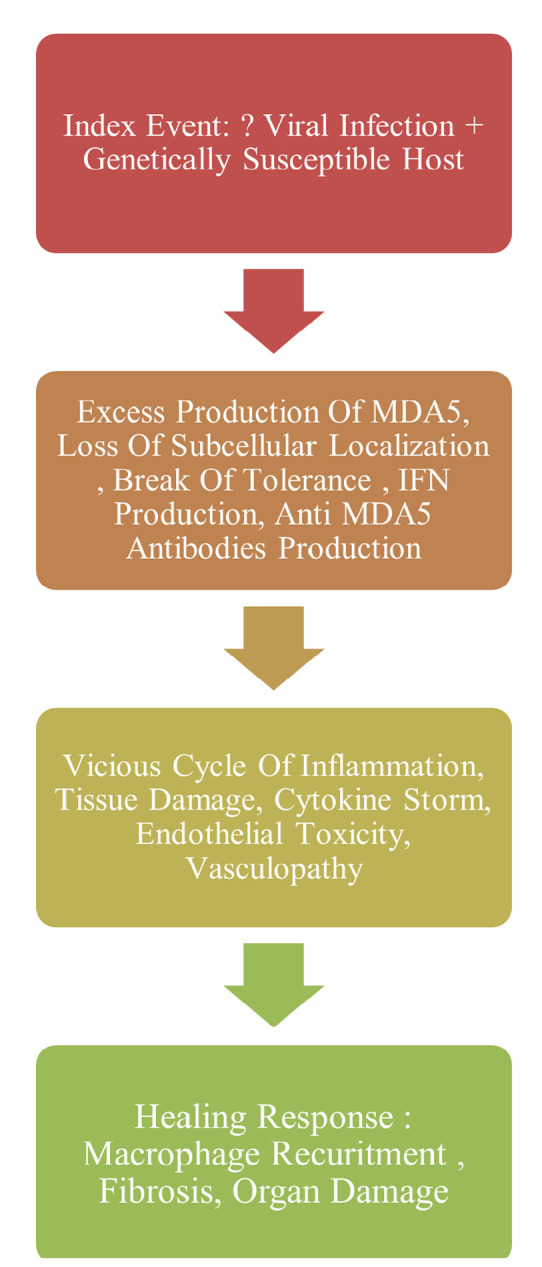
Pathogenesis of MDA 5 associated inflammatory disorders.

MDA5 forms the origin of the proinflammatory and interferon-generating feedback loop. MDA5 was initially isolated in melanoma cells and is an antiviral pattern recognition cytosolic receptor that predominantly recognises long-stranded dsRNA released from RNA viruses, DNA viruses, and mitochondria. After binding to dsRNA and interacting with mitochondrial antiviral signalling protein (MAVS), MDA5 enhances the transcription of interferon-dependent (IFN) genes. At one end of the spectrum generated anti-MDA5 antibodies tend to inactivate MDA5, impairing antiviral responses and promoting multisystemic inflammation to enhance viral clearance which produces more damage to the host cell. On the other end, anti-MDA5 antibodies might lead to persistent activation of MDA5 producing excess interferon and a hyperinflammatory state. Other mechanism includes the formation of immune complexes, cell penetration with downstream pathway disruptions, and antibody-dependent cytotoxicity^3^ (**Figure 8**). Among IgG subtypes anti-MDA5 IgG-1 and IgG3 antibodies are associated with RPILD and death. IgA anti-MDA5 are commonly identified with unclear significance and IgM isotypes are unusual.^4^

**Figure 9.**
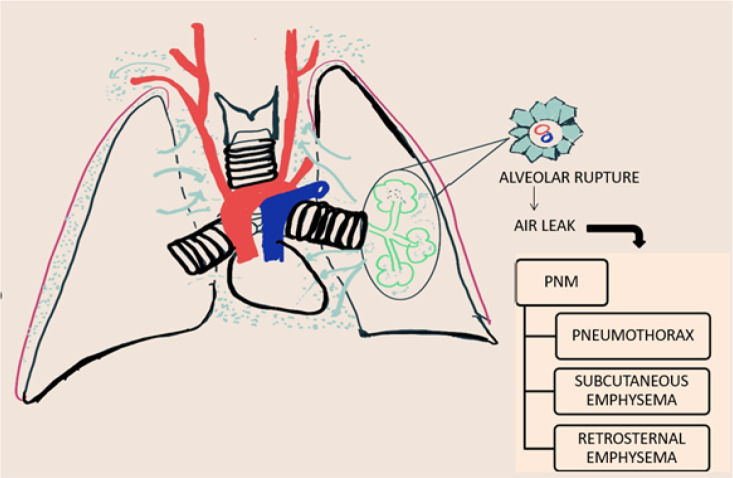
Pathogenesis of pneumomediastinum: Macklin Effect.

**Figure 10.**
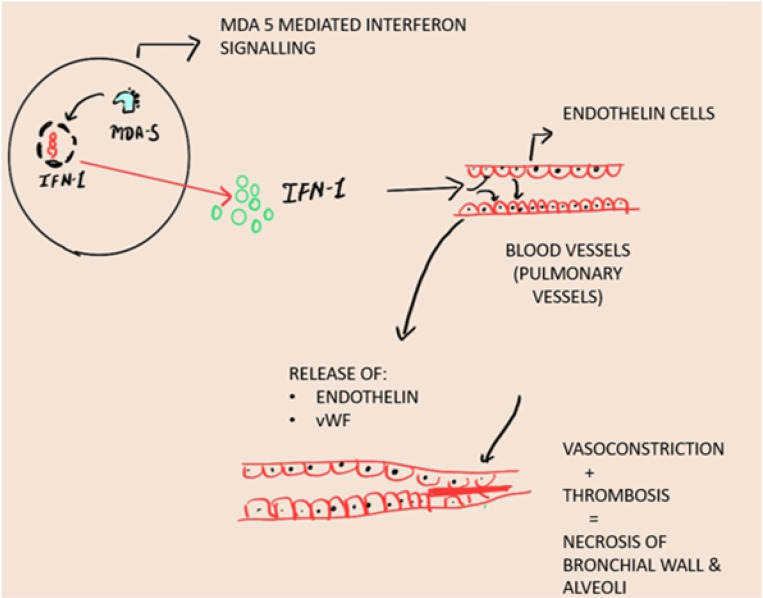
Pathogenesis of pneumomediastinum. Vasoconstriction and thrombosis induce ischemia leading necrosis to alveolar tissue, making it prone to spontaneous rupture.

The proposed mechanism of spontaneous pneumomediastinum in anti-MDA5+ dermatomyositis involves a rise in interalveolar pressure. A rise in pressure leads to alveolar rupture. The air thus gets diffused to the pulmonary interstitium, subcutaneous tissue, and mediastinum. This process is called as spontaneous PNM Macklin effect. SPN is seen more frequently in patients with underlying parenchymal lung disease or ILD as lung architecture is destroyed secondary to inflammatory cell infiltration and alveolar walls become fragile. Thus, factors causing alveolar hyperinflation such as a severe cough, asthma, pulmonary function test, ILD exacerbation, etc., can lead to PNM. In patients with severe ILD, subpleural or paracardial blebs have also been reported due to the distortion of lung architecture, and the rupture of paracardial blebs may also lead to air leakage in the mediastinum^8^. Another possible explanation is alveolar tissue necrosis and rupture following ischemia induced by DM-related vasculopathy which is characterised by endothelium damage and endothelin release. It leads to localised vasoconstriction and focal ischemia. Von Willebrand factor (vWF) is upregulated in MDA5+ DM causing thrombosis and ischemia. Glucocorticoids are thought to interfere with protein metabolism and potentially weaken the pulmonary interstitium. Pneumomediastinum usually occurs in association with ILD or other parenchymal changes however, it has also been reported in patients with no underlying abnormality and tends to have a favourable prognosis (**Figures 9–10**).

Based on clinical features, three phenotypes have been identified: 1. ‘rheumatoid cluster’ – predominant arthritis and cutaneous manifestations, infrequent RP-ILD, seen in females and has overall good prognosis; 2. ‘vasculopathy DM cluster’: characterised by vasculopathy, Raynaud’s phenomenon, skin ulcers, typical dermatomyositis rashes, and myositis; more common in males, intermediate risk of RP-ILD, and favourable prognosis; and 3. ‘RP-ILD cluster’ with poor prognosis, very high rates of RP-ILD and death.

Clinical features include heliotrope rash, periorbital eyes, facial erythema, V-sign (erythema on anterior chest), shawl sign (erythema on shoulders and back), Gottron’s papules, Gottron’s sign, and mechanic’s hand. MDA5+ DM patients are predisposed to RP-ILD, PNM, macrophage activation syndrome (MAS), and spontaneous intramuscular haemorrhage (SIH), which are rare complications, but potentially lethal. The estimated odds ratio is 15.8 for the occurrence of PNM in MDA 5 DM patients. It is frequently seen in patients with cutaneous ulcers and RP-ILD. Patients with PNM have chest pain, dyspnoea, neck pain, and dysphagia, which is often accompanied by subcutaneous emphysema and pneumothorax. On examination, a churning sound with every heartbeat can be heard, called Hamman’s Sign. PNM is more commonly found in patients having vasculopathic manifestations. Cutaneous vasculopathy could relate to either an endothelial inflammatory process or coagulation-fibrinolytic system dysfunction which contributes to the pathogenesis of PNM in MDA5 DM patients.^5^ Several studies have tried to identify the factors pre-disposing to the development of PNM some of which include RP-ILD, elevated ferritin, anti-RO 52 antibody positivity, anti-MDA 5 positivity along with titres which can be used for diagnosis as well as predicting progression, older age, high neutrophil-lymphocyte ratio (NLR), lymphopenia, elevated lactate dehydrogenase (LDH), and fever. The high titre of anti-MDA5 antibody is associated with acute phase or poor outcome while patients with low titre have a relatively better prognosis.^6^

Two important risk factors- “slow air leak” defined as time to progression of dyspnoea (newly emerged respiratory failure, mechanical ventilation required, or decrease in PaO2 >30 mmHg after PNM) >3 days and “early initiation of immunosuppressive agents” defined as initiation of immunosuppressive agents within 1 month following steroid therapy, were associated with decreased risk of PNM. Time taken for the progression of dyspnoea in PNM patients is related to the size and persistence of air leak. Hence, delay of >3 days might indicate a smaller defect and thus, a better prognosis. In a prospective study higher serum albumin level was revealed to be a protective factor.^7^ Elevated C-reactive protein (CRP, > 1 mg/dL), KL-6 (> 1000 ng/dL), decreased T helper subsets and serum CD 206 identify patients with a poor prognosis and at high risk for mortality.

**Table 1.** Comparison of patients’ clinical and biochemical characteristics.

	**Case 1**	**Case 2**	**Case 3**
**Age at presentation**	52 years	50 years	18 years
**Gender**	Female	Male	Female
**Disease duration**	1 month	2 months	9 months
**Skin lesions**	Heliotrope rash	Inverse Gottron signUlcers over elbows and feet	Periorbital oedema, Erythematous crusting, non-scarring alopecia, Flagellate erythema
**MMT8 at presentation**	64/80	80/80	50/80
**Haemoglobin**	13.2 g/dL	14.5 g/dL	9.5 g/dL
**NLR**	3050/1000 = 3.05	10500/1900 = 5.5	4000/1650 = 2.42
**Lymphopenia**	Absent	Absent	Absent
**SGOT/SGPT**	89/181 U/L	27/25 U/L	429/162 U/L
**Creatinine**	0.40 mg/dL	0.53 mg/dL	0.8mg/dL
**CRP**	8.64mg/dL	1.83mg/dL	1mg/dL
**Ferritin**	5720 ng/ml	852 ng/ml	400ng/ml
**CK NAC**	564 U/L	30 U/L	35U/L
**LDH**	506 U/L	583 U/L	600U/L
**CT Chest Pneumomediastinum**	Yes		Yes
**Pneumothorax**	Yes	No	No
**ANA**	Nuclear granular	NA	Negative
**Myositis profile**	MDA5+	MDA5+ Ro 52+	MDA5+
**ENA profile**	Ku+, Ro+	Ro52 3+	Ro52 3+
**Treatment**	Cyclophosphamide Rituximab Tacrolimus IVIG	Cyclophosphamide Tacrolimus	Methotrexate
**Outcome**	Death	Death	Improved

In a case series by Le Goff et al., 11 patients of myositis were included comprising 8 patients of DM, 2 cases of PM, and 1 case of sclerodermatomyositis. The mean age of the patient was 42 years (range 21–75 years), predominantly females, 55.6% of DM patients were clinically amyopathic, and the diagnosis was made at a mean of 5 months after the first symptom onset. In this case, series lesser number of patients had cutaneous manifestations, and all had underlying ILD. The factors associated with poor survival observed in this study were the absence of muscle weakness, an initial decrease in vital capacity, or carbon monoxide diffusion capacity before the onset of pneumomediastinum. The use of high-dose methylprednisolone infusions was associated with a poor outcome.^8^ In another study, initial serum ferritin, P[A-a]O2, and right middle lobe ground glass opacity (GGO) score were found to significantly relate to death. Survival rates after 24 weeks were significantly lower among patients with an initial ferritin level of ≥450 ng/mL, P[A-a]O2 of ≥30 mmHg, and a right middle lobe GGO score of ≥2.^9^ As per a study conducted by Liu T et al., NLR in patients of anti-MDA5+ DM was significantly higher in non-survivors than survivors (p < 0.001). Lactate dehydrogenase and C-reactive protein were significantly increased when NLR was greater than 4.86. Results of multivariate analysis established that NLR > 4.86 was an independent predictor of mortality.^10^

In the 3 cases we described, all had cutaneous manifestations and 1 had amyopathic presentation. Patients with lag in the development of pneumomediastinum from symptoms onset and younger age had better prognosis whereas patients with higher ferritin, elevated CK NAC, higher NLR, high CRP, rapid development of pneumomediastinum and extensive ILD had poor prognosis, similar to what has been described in previous study. In patients with normal lung parenchyma, there is a tendency to develop pneumomediastinum, but the overall prognosis is good.

There are no standard treatment guidelines for the management of pneumomediastinum associated with CTDs. Supportive therapy like oxygen supplementation, cough suppressants, and vaccination must be used in all patients invariably. Early aggressive immunosuppressive therapy is the mainstay treatment of MDA5+ DM-associated PNM, as most cases are secondary to underlying ILD. Non-invasive positive pressure ventilation (NPPV) should not be used as it has been shown to aggravate mediastinal emphysema through inappropriate outflow of air. Early mechanical ventilation would be a preferable option to improve PNM’s anoxic state. Usually, triple therapy i.e., glucocorticoids, calcineurin inhibitors, and INJ cyclophosphamide are initiated in patients with RP-ILD. JAKi have been tried in early cases. Rituximab, PLEX, IVIG, and surgical options are reserved for refractory cases. ECMO can be used as a bridge therapy. In some cases when patients have not improved despite all measures, they have taken up lung transplants with good outcomes. Hence, lung transplants can be used as a rescue therapy.

## CONCLUSION

Patients with MDA5+ DM have more tendency to develop PNM with morbidity estimated to be 15% and mortality around 60% maximally seen within 1 month of diagnosis. Though rare but occurrence of PNM could be practically lethal hence clinicians should have high suspicion and should always actively search for poor prognostic markers including older age, high NLR, lymphopenia, elevated LDH, hyperferritinaemia, fever, elevated CRP, amyopathic presentation and baseline severity of the ILD. There are no standard guidelines for the management of PNM. Data is available only through case reports, case series, or cohort studies nonetheless, corticosteroids and immunosuppressive treatment should be initiated as soon as possible, particularly in the presence of poor prognostic factors as it seems to be the only way to attain a favourable outcome.^1,5^
